# Cepharanthine improves blood glucose levels and mitigates renal injury in streptozotocin-induced diabetic rats via restoring pancreatic β-cell integrity and reducing inflammation

**DOI:** 10.3389/fphar.2026.1807706

**Published:** 2026-06-19

**Authors:** Mohamed A. Katary, Yara A. Samra, Karim M. Saad, Kim Capehart, Rafik Abdelsayed, Ahmed A. Elmarakby

**Affiliations:** 1 Department of Oral Biology, Dental College of Georgia, Augusta University, Augusta, GA, United States; 2 Department of Pharmacology & Toxicology, Faculty of Pharmacy, Damanhour University, Damanhour, Egypt; 3 Department of Biochemistry, Faculty of Pharmacy, Mansoura University, Mansoura, Egypt; 4 Department of Pharmacology & Toxicology, Faculty of Pharmacy, Mansoura University, Mansoura, Egypt; 5 Department of General Dentistry, Dental College of Georgia, Augusta University, Augusta, GA, United States; 6 Department of Pathology & Diagnostic Sciences, Dental College of Georgia, Augusta University, Augusta, GA, United States

**Keywords:** blood glucose, cepharanthine, endothelial dysfunction, insulin secretion, oxidative stress, PDX-1, renal inflammation, streptozotocin

## Abstract

**Background/Objectives:**

We have previously demonstrated that the natural alkaloid Cepharanthine (Ceph) reduces renal injury in streptozotocin (STZ)-induced insulin-deficient diabetic rats via antioxidant and anti-inflammatory mechanisms. However, the hypoglycemic mechanism of Ceph remains unclear. We hypothesize that Ceph reduces blood glucose in STZ-induced insulin-deficient diabetic rats via restoring pancreatic β-cells function and insulin secretion.

**Methods:**

Diabetes was induced in male Sprague-Dawley rats by a single intraperitoneal injection of STZ (50 mg/kg, i. p). Rats with blood glucose ≥250 mg/dL were confirmed to be diabetic. Rats were randomly assigned to four groups (n = 6/group): Control, Control + Ceph, Diabetic, and Diabetic + Ceph. Rats were treated with either vehicle or Ceph (10 mg/kg/day, i. p) for 6 weeks. Pancreatic β-cell integrity was evaluated using TUNEL assay and pancreatic duodenal homeobox 1 (PDX-1) immunofluorescence. Aortic rings vascular reactivity was assessed using wire myography, and renal function was determined by measuring creatinine clearance, albuminuria, and podocalyxin excretion. Renal inflammation and fibrosis were quantified via ELISA assessment of ICAM-1 and TGF-β and Masson’s trichrome staining.

**Results:**

Ceph treatment significantly reduced blood glucose levels and increased plasma insulin levels in diabetic rats. The % TUNEL^+^ cells were significantly elevated in the pancreas of diabetic rats, and Ceph treatment reduced these changes. Immunofluorescence analysis also revealed an increase in PDX-1 expression levels in Ceph treated vs. non treated diabetic rats. Ceph improved vascular endothelial function as it significantly attenuated the impairment in aortic ring relaxation to acetylcholine in diabetic rats. Moreover, Ceph significantly reduced markers of renal injury as evidenced by the reduction in albuminuria and podocalyxin excretion levels and the improvement in creatinine clearance in diabetic rats. Ceph also reduced renal oxidative stress, inflammation, and fibrosis as evidence by the reduction in Thiobarbituric Acid Reactive Substances (TBARs) excretion, and renal ICAM-1 and TGF-β levels in diabetic rats, respectively.

**Conclusion:**

These data suggest that Ceph halts the progression of diabetic nephropathy in rats not only via its antioxidant and anti-inflammatory properties but also via the reduction in pancreatic β-cells apoptosis and increased β-cells integrity in STZ-induced insulin-deficient diabetic male Sprague-Dawley rats.

## 1 Introduction

Diabetes mellitus is a major global health problem and a leading cause of morbidity and mortality. This is due to its strong association with cardiovascular disease (CVD) and chronic microvascular complications such as nephropathy, neuropathy and retinopathy (Einarson et al., 2018). Hyperglycemia is a central driver of diabetic kidney disease by promoting glomerular and tubular injury which leads to proteinuria and progressive loss of renal function (Elmarakby and Sullivan, 2012; Jin et al., 2023). In multiple cohorts, one-third of patients with diabetes are known to develop diabetic nephropathy, which often progresses to end-stage renal disease (ESRD) in ∼50% of patients within 10 years (AyodeleAlebiosu and Salako, 2004; Shahbazian and Rezaii, 2013). Diabetic nephropathy the leading cause of ESRD in many regions, where kidney transplantation becomes the only treatment option (Rout and Jialal, 2025).

Our group and others have demonstrated that hyperglycemia-induced renal oxidative stress, inflammation and fibrosis are key mechanistic pathways that accelerate progression of diabetic nephropathy and therefore represent important therapeutic targets to slow the progression of diabetic nephropathy and prevent further decline in renal function (Elmarakby et al., 2010; Elmarakby et al., 2012; Elmarakby and Sullivan, 2012). Emerging therapies targeting these mechanisms such as endothelin receptor antagonists, mineralocorticoid receptor antagonists, and antifibrotic agents have shown encouraging results in slowing diabetic kidney disease progression (Liu and Liu, 2020; Ruiz-OrtegaLamas and Ortiz, 2022). Likewise, sodium-glucose cotransporter 2 (SGLT2) inhibitors provide renal protection through metabolic and hemodynamic effects, although concerns remain regarding volume depletion and subsequent dehydration with chronic use of SGLT2 inhibitors (Garofalo et al., 2019; Yau et al., 2022). In parallel, vascular complications are a major cause of mortality in diabetes, with endothelial dysfunction driven by oxidative stress and impaired nitric oxide (NO)/cyclic guanosine monophosphate (cGMP) signaling playing a role in diabetic vasculopathy (Koren et al., 2025). Thus, pharmacological agents that target both renal and vascular oxidative injury may also offer comprehensive protection in diabetes.

Cepharanthine (Ceph), a biscoclaurine alkaloid isolated from *Stephania cephalantha Hayata,* has been clinically used in Japan since the 1950s and is registered for human use, primarily for conditions such as radiation-induced leukopenia and alopecia (Bailly, 2019). Ceph is known to have multiple pharmacological effects including but not limited to anti-oxidant, anti-inflammatory, anti-cancer, anti-viral, immune-modulatory and anti-parasitic properties (Liu et al., 2023). Moreover, Ceph is relatively inexpensive, and clinical utilization of Ceph has demonstrated tolerability and safety in long-term use (Rogosnitzky and Danks, 2011). Studies have previously shown that Ceph protects against diabetic renal injury by suppressing oxidative stress, fibrosis, NF-κB activation, and NLRP3 inflammasome signaling in streptozotocin (STZ)-induced insulin-deficient diabetic rats. And these changes were associated with decreases in blood glucose levels (Samra et al., 2016). These findings suggest a role of Ceph in preserving pancreatic β-cell function and insulin secretion. Therefore, the current study was designed to explore the metabolic, vascular, and renal protective mechanisms of Ceph in STZ-induced insulin-deficient diabetic male rats, with particular focus on its effects on insulin levels, pancreatic β-cell integrity, inflammation, and endothelial function.

## 2 Materials and methods

### 2.1 Animals

*A priori* power analysis was conducted using G*Power software (Version 3.1.9.7; University of Kiel, Germany) and it was estimated that n = 6 rats per group would provide 80% power (α = 0.05) to detect a 50% difference in renal inflammation and injury. Rat procedures were performed in accordance with the Public Health Service Guide for the Care and Use of Laboratory Animals and Augusta University guidelines (Protocol #2012–0,426). Twenty-four male Sprague Dawley rats (11–12-week-old) were obtained from Envigo Inc. (Indianapolis, IN). Rats were pathogen-free and experimentally naïve without any previous procedures. Rats were housed under controlled conditions (72°F–74°F, 12 h light/dark cycle, light was turned on at 6:00 a.m. and tuned off at 6:00 p.m. daily) with *ad libitum* access to standard chow and water. Rats were acclimatized for 1 week prior to experimental procedures. To minimize potential confounders, rats from different experimental groups were housed in separate cages but maintained under identical environmental conditions. Cage positions within the animal facility were rotated weekly to minimize location-related bias, and all treatments and measurements were conducted at the same time of the day (9am–11am).

### 2.2 Treatment procedures and sample collection

Rats and cages were assigned unique identification numbers prior to the start of the experiment. Rats were first randomly allocated to either a non-diabetic or diabetic group using an automated random number generator. Insulin-deficient diabetes was induced by a single intraperitoneal injection of streptozotocin (STZ; 50 mg/kg, i. p) freshly dissolved in 0.1 M citrate buffer (pH 4.5) (Sigma, St. Louis, MO), while non-diabetic rats received vehicle alone. The dose of STZ was selected to induce a stable model of insulin-deficient diabetes with consistent hyperglycemia while minimizing non-specific systemic toxicity and higher rats mortality rate, consistent with established protocols (Ghasemi and Jeddi, 2023). Fasting blood glucose levels were measured daily for three consecutive days following STZ administration using a OneTouch Ultra glucometer (Life-Scan, Malvern, PA) via tail vein sampling. Rats with blood glucose levels ≥250 mg/dL at 72 h post-STZ injection were considered diabetic and included in the study.

After confirmation of diabetes, rats within each group (diabetic and non-diabetic) were randomly reallocated using an automated random number generator to receive either cepharanthine (Ceph; 10 mg/kg/day, i. p, Fisher scientific, St. Paul, MN) or vehicle, resulting in four experimental groups (n = 6 per group): control untreated, control + Ceph, diabetic untreated, and diabetic + Ceph. Group allocation was known to the investigator administering treatments; however, investigators performing outcome assessments and data analysis were blinded to groups assignment. Ceph was dissolved in phosphate-buffered saline (PBS) containing 0.5% carboxymethyl cellulose and dose was selected based on our previous findings that showed the effectiveness of the selected dose to reduce markers of renal inflammation and injury in STZ-induced insulin-deficient diabetic rats (Samra et al., 2016).

At the end of the 6 weeks treatment, rats were placed in metabolic cages (Nalgene Corp, Rochester, NY) for 24 h urine collection before rats were anesthetized with isoflurane to collect blood, pancreas, aorta and kidneys. One kidney was used for histopathological assessments, and another kidney was snap frozen in liquid nitrogen for later assessment of renal transforming growth factor-beta (TGF-β) and intercellular adhesion molecule-1 (ICAM-1). We determined kidney to body weight % as an indicator of kidney enlargement and compensatory renal hyperfiltration associated with diabetes. No rats were excluded from the study, and no adverse events were observed.

### 2.3 Quantification of plasma insulin, cholesterol, TBARs, cGMP, TGF-β and ICAM-1

Plasma was isolated from collected blood (using 5 mM EDTA as an anticoagulant) and used to assess insulin and cholesterol levels using commercially available kits from (Millipore, Billerica, MA) and (Wako Diagnostics, Richmond, VA), respectively. Plasma TBARs and cGMP levels were also assessed using commercially available kits from Cayman Chemical, Ann Arbor, MI as markers of systemic oxidative stress and nitric oxide (NO) bioavailability, respectively. Plasma TGF-β and ICAM-1 levels were measured as markers of systemic inflammation using commercial ELISA kits from R&D System Inc., Minneapolis, MN.

### 2.4 Quantification of plasma and urinary creatinine, urinary albumin, podocalyxin, and TBARs

Urine was used to assess markers of renal injury. Urinary albumin and podocalyxin excretion levels were determined using ELISA kits from Biocompare, San Francisco, CA. Urinary TBARs excretion levels were assessed as a marker of renal oxidative stress, using a kit from Cayman Chemical, Ann Arbor, MI. Plasma and urinary creatinine excretion levels were also assessed using a kit from Cayman Chemical, Ann Arbor, MI and used to calculate creatinine clearance as shown previously (Saad et al., 2024).

### 2.5 Assessment of vascular reactivity in aortic rings

Thoracic aorta was used to assess vascular function. The aorta was isolated, cut into 2 mm rings and mounted on pins for isometric myography (Danish Myo Technology A/S, Aarhus, Denmark) in chambers filled with aerated (95% O2 and 5% CO2) Krebs buffer (130 mM NaCl, 4.7 mM KCl, 1.17 mM MgSO_4_, 14.9 NaHCO_3_, 5.6 mM Dextrose, 1.56 mM CaCl_2_.2H_2_O, 0.03 mM EDTA) heated to 37 °C. The tension was adjusted to 30 mN and aortic rings were allowed to equilibrate for 30 min with the Krebs buffer replaced every 15 min before the viability of the vessel was determined utilizing 10^-6^ M phenylephrine (PE) as vasoconstrictor followed by vasorelaxation to 10^-6^ M acetylcholine (Ach). Only aortic rings that relaxed at least 80% of the maximal PE-induced contraction were included in the study. Aortic endothelial-dependent relaxation was assessed by performing cumulative concentration response (CCR) to Ach (10^-9^–10^-4^ M) after vessel pre-constricted with PE (10^-6^). Aortic endothelial-independent relaxation was also assessed by performing cumulative concentration response (CCR) to sodium nitroprusside (SNP, 10^-9^–10^-4^ M) after vessel pre-constricted with PE (10^-6^).

### 2.6 Assessment of β-cells histopathology, pancreatic and duodenal homeobox 1 (PDX1) and β-cells apoptosis

To further confirm that Ceph improves glycemic status in STZ-induced insulin-deficient diabetic rats, we stained pancreatic sections with hematoxylin and eosin (H&E) to assess histological changes in pancreatic morphology with the help of Dr. Abdelsayed in blind fashion. Pancreas was fixed in formalin before being paraffin embedded for histopathological assessments. Pancreatic sections were deparaffinized using xylene and serial alcohol dilution then stained with H&E staining. Additional pancreatic sections were also deparaffinized before Terminal dUTP nick end-labeling (TUNEL) assay was performed utilizing one-step TUNEL *In Situ* Apoptosis Kit (Green, FITC) from Elabsciences, Houston, TX. Green fluorescence was used as an indicator of DNA damage and apoptotic cell death whereas 4′,6-diamidino-2-phenylindole (DAPI) was utilized as a blue-fluorescent nuclear stain. We also assessed pancreatic and duodenal homeobox 1 (PDX1) expression levels in pancreatic sections as a marker of pancreatic development and maturation using immunofluorescence technique. Briefly, deparaffinized pancreatic sections were incubated overnight at 4 °C with anti-PDX1 (Santa Cruz Biotechnology, Dallas, TX) primary antibodies. On the second day, washing and blocking procedures were repeated before incubation of slides for 1 h in Oregon green-labeled secondary antibodies (Molecular Probes, Carlsbad, CA) (DAPI) was utilized as a blue-fluorescent nuclear stain, Images were obtained using (Axio Imager 2, Carl Zeiss) with 100x magnification. % of TUNEL^+^ cells or PDX1 expression were determined using multipoint selection tool in ImageJ software (n = 4/group).

### 2.7 Assessment of renal Masson’s trichrome and collagen IV expression

Kidney sections from different rat groups (n = 4 rats/group) were stained with Masson’s trichrome to assess the amount of collagen deposition. Ten microscopic images of the kidney sections per rat were randomly taken at 200X magnification using Axio Imager 2, Carl Zeiss microscope and scoring of slides was performed blindly and graded on a scale of 1–5. Kidney sections were also used for immunohistochemical assessment of kidney collagen IV (Novus Biologicals, Centennial, CO). Briefly, slides were deparaffinized with xylene and serial alcohol dilution. Citrate buffer was used for antigen retrieval and hydrogen peroxide (1%) was then used to quench kidney peroxidase followed by incubation with the collagen IV primary antibodies overnight at 4 °C. Slides were incubated with proper secondary antibodies for 90 min before streptavidin-HRP (Vector laboratories, Newark, CA) was added for 30 min followed by addition of substrate chromogen AEC and hematoxylin as a counterstaining (Vector laboratories, Newark, CA). Images were captured using Axio Imager 2, Carl Zeiss microscope and the collagen IV expression area % was assessed in captured images at ×200 magnification using ImageJ software.

### 2.8 Statistical analysis

All data are presented as mean ± SEM. Normality of data distribution was assessed using the Shapiro–Wilk test. All datasets met the assumptions of normality and equal variance and were therefore analyzed using one-way analysis of variance (ANOVA) followed by Tukey’s *post hoc* test for multiple group comparisons except for Masson’s trichrome fibrosis scoring, which did not follow a normal distribution and was analyzed using Kruskal–Wallis test followed by Dunn’s multiple comparisons test. Differences were considered statistically significant with *p* < 0.05 compared to the control rat groups. Analyses were performed using Graph Pad Prism Version 10.0 software.

## 3 Results

### 3.1 Effect of Ceph on blood glucose, plasma insulin and cholesterol levels, and kidney to body weight ratio in STZ-induced insulin-deficient diabetic rats

**FIGURE 1:**
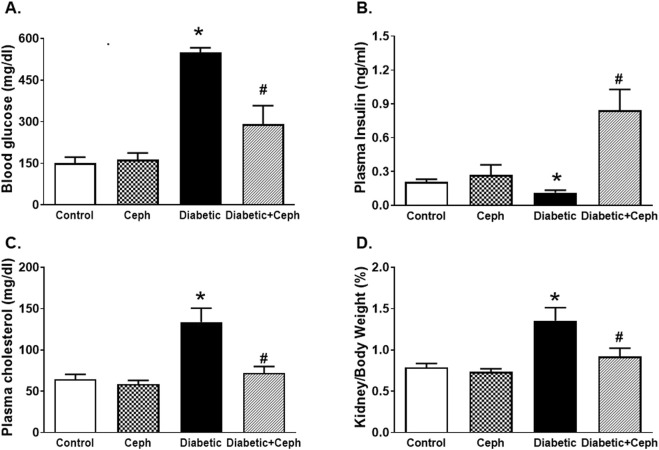
Effect of Cepharanthine (Ceph) treatment on random blood glucose, plasma insulin and cholesterol levels and kidney/body weight ratio in male control and streptozotocin (STZ)-induced insulin deficient diabetic rats. Male Sprague Dawley rats (11–12 weeks/old) were randomized to receive single i.p injection of citrate buffer or STZ (50 mg/kg). Control and diabetic rats were then received daily i.p Injection of vehicle control (phosphate buffer saline containing 0.5% carboxymethyl cellulose) or Cepharanthine (Ceph) (10 mg/kg body weight, i.p p daily) for 6 weeks. Blood glucose **(A)**, plasma insulin **(B)**, plasma cholesterol **(C)** and kidney to body weight % **(D)** were assessed at the end of 6 weeks of Ceph treatment. Values were expressed as means ± SEM (n = 6 per group). ^∗^ Indicates significant difference when compared with the control or Ceph group and ^#^ indicates significant difference when compared with the diabetic group at p < 0.05.

After injection of STZ or vehicle and before starting Ceph treatment**,** average fasting blood glucose levels in vehicle injected control rats were 102.7 ± 5.3 mg/dL whereas average fasting blood glucose levels were 414.4 ± 61.6 mg/dL in STZ-injected diabetic rats. After 6 weeks of either vehicle or Ceph treatment, induction of diabetes with STZ injection significantly increased random blood glucose level compared with control rats with or without Ceph treatment (P < 0.0001, Figure 1A). Ceph treatment significantly reduced the elevation in plasma glucose levels in diabetic rats (P < 0.0001, Figure 1A). The elevation in blood glucose in STZ injected rats was associated with decreases in plasma insulin levels (P < 0.008) and Ceph treatment restored plasma insulin levels in diabetic rats (Figure 1B, P < 0.002). Ceph treatment also significantly reduced the elevation in plasma cholesterol levels in diabetic rats (Figure 1C, P < 0.003). Induction of diabetes with STZ significantly decreased body weight vs. control untreated or Ceph treated rats (average body weight was 287 ± 16.1 grams in diabetic rats vs. 396.6 ± 10.9 grams in untreated control rats or 394.2 ± 8.2 grams in Ceph treated control rats, P < 0.05) Ceph treatment tended to improve body weight loss in diabetic rats, although these changes did not reach statistical significance (305.9 ± 7.8 gram, P < 0.1), however body weight remained significantly lower than control rats with or without Ceph treatment (P < 0.05). Diabetic rats showed significant increase in kidney to body weight % compared to control rats (P < 0.003), however Ceph treatment significantly mitigated this increase in kidney to body weight % in diabetic rats (Figure 1D, P < 0.03).

### 3.2 Effect of Ceph on STZ-induced changes in pancreatic β-cells architecture, apoptosis and maturation

**FIGURE 2:**
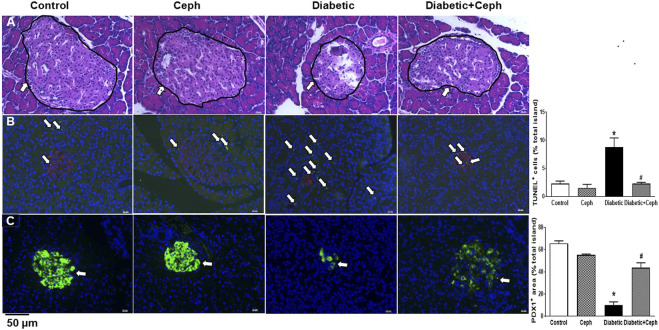
Effects of Cepharanthine (Ceph) treatment on Pancreatic β-cells morphology, apoptosis and development in control and STZ-induced insulin deficient diabetic rats. Representative images of pancreatic sections stained hematoxylin and eosin (H & E) showing pancreatic β-cells histoarchitecture **(A)**, TUNEL staining (green) as an indicative of pancreatic apoptosis **(B)** utilizing DAPI and nuclear staining (blue) and immunofluorescence staining of pancreatic and duodenal homeobox 1 (PDX1) expression **(C)** as a marker of pancreatic development and maturation in control and diabetic rat with or without Ceph treatment for 6 weeks at 100X magnification. % of TUNEL^+^ cells or PDX1 expression were determined using multipoint selection tool in ImageJ software (n = 4/group). ^∗^ Indicates significant difference when compared with the control or Ceph rat group and ^#^ indicates significant difference when compared with the diabetic rats at p < 0.05.

Utilizing Dr. Abdelsayed pathological expertise in a blind fashion as shown previously (Elmarakby et al., 2011), representative images of pancreatic sections from control or Ceph rat groups showed normal histoarchitecture with well-developed globules of acini and normal pancreatic islet cells and no clear evidence of fibrosis or inflammation (Figure 2A). As highlighted in black color, images from pancreatic section of diabetic rats showed atrophy of islet cells and vacuolation in addition to inflammatory edema (Figure 2A). Images from Ceph treated diabetic rats revealed well-regenerated pancreatic cells with normal pancreatic islet cells and prominent nucleus with minimum inflammatory edema (Figure 2A). Utilizing TUNEL assay, we assessed apoptotic cell death in pancreatic β-cells. As shown in Figure 2B, % of TUNEL^+^ cells (green fluorescence) increased in diabetic rats (P < 0.002) and % of TUNEL^+^ cells were reduced by Ceph treatment (P < 0.002). It is worth mentioning that red fluorescence in the representative images indicated insulin, however we did not quantify it since we already measured plasma insulin levels as shown in Figure 1B in our rat groups. Ceph treatment restored the decrease in pancreatic insulin secretion in diabetic rats which is consistent with plasma insulin data. We further assessed PDX1 expression level in pancreatic sections, as a master regulator of pancreatic function. As shown in Figure 1C, % of PDX-1 expressions in pancreatic sections (green fluorescence) significantly decreased in diabetic rats (P < 0.0001) and Ceph treatment restored this decrease (P < 0.0001).

### 3.3 Effect of Ceph on STZ-induced systemic oxidative stress and aortic vascular endothelial dysfunction

**FIGURE 3:**
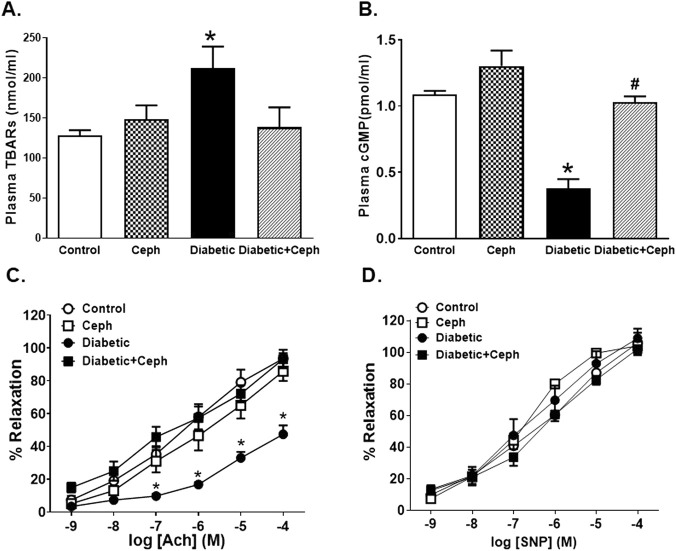
Effects of Catharanthine (Ceph) treatment on systemic oxidative stress and vascular endothelial function in aortic rings isolated from control and STZ-induced insulin deficient diabetic rats. Plasma Thiobarbituric Acid Reactive Substances (TBARs, **(A)** and cGMP **(B)** levels were assessed as markers of systemic oxidative stress and nitric oxide (NO) bioavailability, respectively in male control and diabetic rat with or without Ceph treatment. Values were expressed as means ± SEM (n = 6 per group). ^*^Indicates significant difference from control or Ceph rat groups and ^#^ indicates significant difference from diabetic rats at p < 0.05. Aortic endothelial-dependent relaxation was assessed by performing cumulative concentration response to acetylcholine (Ach) after vessel pre-constricted with phenylephrine **(C)** whereas endothelial-independent relaxation was also assessed by performing cumulative concentration response to sodium nitroprusside (SNP) after vessel pre-constricted with phenylephrine **(D)** in male control and diabetic rat ± Ceph treatment for 6 weeks. Values were expressed as means ± SEM (n = 6 per group). ^∗^ Indicates significant difference vs. corresponding value of either control, Ceph or diabetic + Ceph groups at p < 0.05.

We found that plasma TBARs levels as a marker of systemic oxidative stress were significantly elevated in diabetic rats vs. control or Ceph groups, and this elevation was prevented by Ceph treatment (P < 0.04, Figure 3A). Since NO exerts its physiological effects via activation of soluble guanylyl cyclase to increase production of the second messenger cGMP (Wang et al., 2017), we also assessed plasma cGMP levels as an indicator of NO bioavailability. Consistent with the elevation in oxidative stress, plasma cGMP levels significantly decreased in diabetic rats vs. control or Ceph rat groups (P < 0.0001) and Ceph treatment restored the decrease in cGMP levels in diabetic rats (P < 0.0001, Figure 3B). Since vascular dysfunction is a common hallmark of cardiovascular disease including diabetes (Yang et al., 2024), we assessed changes in vascular function in diabetic rats with or without Ceph treatment. Aortic endothelial-dependent relaxation to Ach was significantly impaired in diabetic rats when compared to either control or Ceph groups and the impairment in aortic endothelial-dependent relaxation to Ach was prevented by Ceph treatment in diabetic rats (Figure 3C, P < 0.001). Induction of diabetes with STZ did not affect smooth muscle function since aortic endothelial-independent relaxation to SNP was not significantly different in diabetic rats with or without Ceph treatment when compared to either control or Ceph rat groups (Figure 3D).

### 3.4 Effect of Ceph on STZ-induced changes in TBARs excretion, albuminuria and creatinine clearance

**FIGURE 4:**
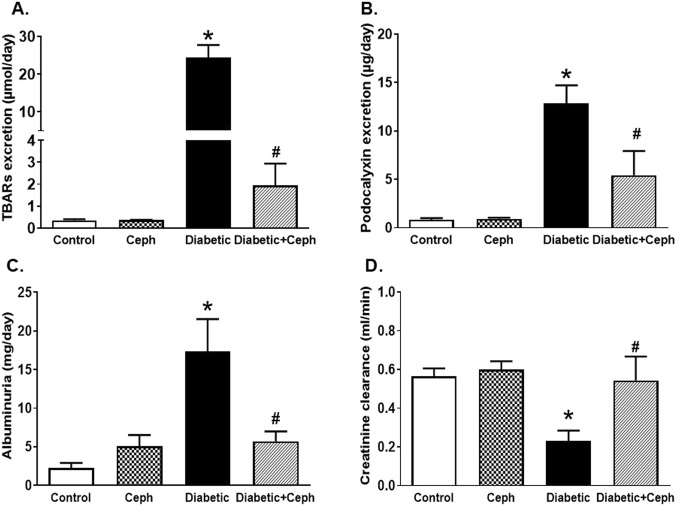
Effects of Cepharanthine (Ceph) treatment on markers of renal oxidative stress and injury in control and STZ-induced insulin deficient diabetic rats. Control and diabetic Rats were placed in metabolic cages for 24 h urine collection after 6 weeks of Ceph treatment to assess urinary TBARs **(A)**, podocalyxin **(B)** and albumin **(C)** excretion levels as well as creatinine clearance **(D)** in control and diabetic male rats ± Ceph treatment (n = 6 per group). ^*^Indicates significant difference from control or Ceph rat groups and ^#^ indicates significant difference from diabetic rat group at p < 0.05.

The elevation in systemic oxidative stress also coincided with elevation in renal oxidative stress since urinary TBARs excretion levels were significantly elevated in diabetic rats (P < 0.0001) and Ceph treatment decreased this elevation (Figure 4A, P < 0.0001). Podocalyxin is a protein found in glomerular epithelial cells and is known to be shed then excreted in urine in conditions associated with glomerular injury (Zeng and Szeto, 2021). Thus, we assessed podocalyxin excretion as an indicative of glomerular injury. As shown in Figure 4B, diabetes significantly increased podocalyxin excretion levels when compared to control or Ceph group (P < 0.002) and Ceph treatment significantly attenuated the elevation in podocalyxin excretion in diabetic rats (P < 0.02). Since diabetes is known to increase renal perfusion and glomerular capillary pressure which in turn leads to protein leakage in urine (Tonneijck et al., 2017), we assessed albumin excretion as markers of renal injury. Albuminuria (Figure 4C) was significantly elevated in diabetic rats vs. control or Ceph group (P < 0.003). Ceph treatment significantly mitigated albuminuria in diabetic rats (P < 0.02, Figure 4C). We also determined creatinine clearance as an indicator of diabetic kidney disease in which diabetes could decrease kidney efficiency to clear waste products such as creatinine from the blood. As presented in Figure 4D, creatinine clearance significantly decreased in diabetic rats vs. control or Ceph group (P < 0.01) and Ceph treatment restored the decrease in creatinine clearance in diabetic rats (P < 0.02).

### 3.5 Effect of Ceph on STZ-induced increases in renal fibrosis and inflammation

**FIGURE 5:**
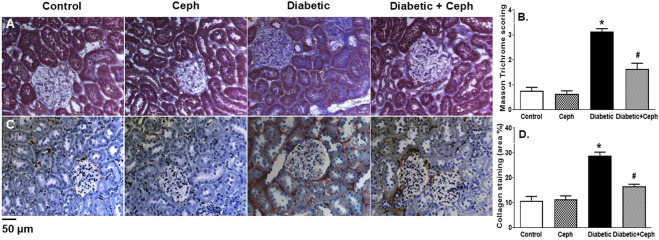
Effects of Cepharanthine (Ceph) treatment on renal histopathological changes and fibrosis in control and STZ-induced insulin deficient diabetic rats. Representative images of renal Masson’s trichrome staining **(A)** and collagen IV expression levels **(B)** in control and diabetic rats ± Ceph treatment for 6 weeks at 200X magnification. Masson’s trichrome scoring **(C)** and % renal collagen IV expression levels **(D)** in control and diabetic male rats ± Ceph treatment for 6 weeks (n = 4/group). ^*^Indicates significant difference from control or Ceph rat groups and ^#^ indicates significant difference from diabetic rats at p < 0.05.

Diabetes is also known to increase renal fibrosis and extracellular matrix (ECM) deposition in the kidney especially in the glomeruli (Ban and Twigg, 2008). Histopathological examination of kidney sections using Masson’s trichrome staining revealed increased renal fibrosis in diabetic rats (blue staining) vs. control or Ceph group (P < 0.0001) and this increase was reduced with Ceph treated diabetic rats (P < 0.0002, Figures 5A,B). We further confirm Masson’s trichrome data for renal fibrosis by immunohistochemical assessment of renal collagen IV expression that is known to increase in glomerular and tubular basement membrane in fibrotic kidney (Ban and Twigg, 2008). Renal collagen IV expression levels were also elevated in diabetic rat vs. control or Ceph group (P < 0.0001), and this effect was significantly mitigated with Ceph treatment in diabetic rats (P < 0.0002, Figures 5C,D).

**FIGURE 6:**
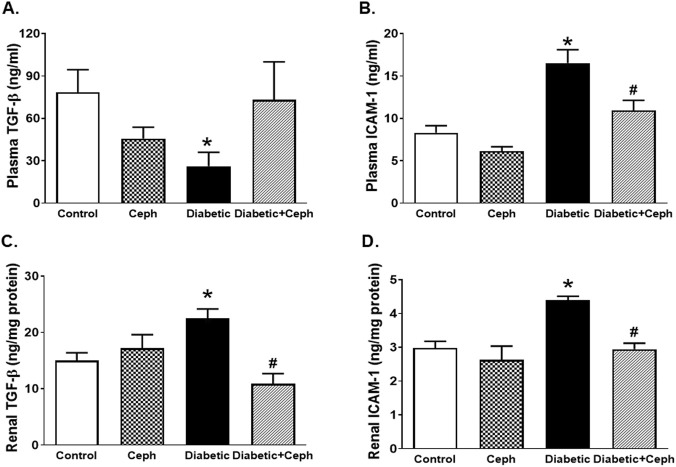
Effects of Cepharanthine (Ceph) treatment on markers of systemic and renal inflammation and fibrosis in control and STZ-induced insulin deficient diabetic rats. Plasma transforming growth factor-beta (TGF-β, **(A)** and intercellular adhesion molecule-1 (ICAM-1, **(B)** levels were assessed as markers of systemic fibrosis and inflammation, respectively in male control and diabetic rat with or without Ceph treatment for 6 weeks. Renal TGF-β and ICAM-1 levels **(C,D)** were also assessed as markers of renal fibrosis and inflammation, respectively in control and diabetic male rats ± Ceph treatment (n = 6 per group. ^*^ Indicates significant difference from control or Ceph rat group and ^#^ indicates significant difference from diabetic rats at p < 0.05.

Previous studies have demonstrated that inflammatory cytokines play an important role in the progression of diabetic renal inflammation and fibrosis (Donate-Correa et al., 2020). Therefore, we assessed the levels of plasma and renal ICAM-1 and TGF-β as markers of systemic and renal inflammation and fibrosis, respectively. Plasma TGF-β was significantly decreased in diabetic vs. control rats (P < 0.008) and Ceph treatment restored this decrease (Figure 6A). Contrary to plasma data, renal TGF-β was significantly elevated in diabetic rats vs. control or Ceph group (P < 0.04) and Ceph treatment reduced this elevation (Figure 6C, P < 0.001). Plasma ICAM-1 also significantly increased in diabetic rats vs. control or Ceph group (P < 0.0002) and Ceph treatment reduced this elevation in diabetic rats (Figure 6B, P < 0.009). Similarly, renal ICAM-1 levels were significantly increased in diabetic rats vs. control or Ceph group (P < 0.009) and Ceph treatment prevented this elevation in diabetic rats (Figure 6D, P < 0.007).

## 4 Discussion

The current study demonstrates that Ceph treatment provides protective effects in STZ-induced insulin-deficient diabetic male rats through improving glycemic control, preserving pancreatic β-islet integrity, and attenuating vascular and renal injury. These findings extend previous work of (Samra et al., 2016), who demonstrated reno-protective effects of Ceph in diabetic rats via modulating NF-κB and NLRP3 inflammasome pathways, along with its antioxidant activity. The current data further reveals that Ceph treatment encompasses direct modulation of pancreatic β-cells apoptosis and regeneration as well as amelioration of STZ-induced endothelial dysfunction.

The selective toxicity of STZ toward pancreatic β-cells underlies its common use in experimental models to induce diabetes-like features characterized by hyperglycemia and reduced insulin production (Ghasemi and Jeddi, 2023). Previous studies have shown that STZ-induced oxidative stress in pancreatic β-cell and activates NF-kB inflammatory pathway, which leads to decreased expression of pancreatic and duodenal homeobox-1 (PDX-1), a transcription factor required for β-cell differentiation and insulin gene transcription (Li et al., 2020). Other studies demonstrated that anti-oxidants maintain β-cell integrity by preventing oxidative stress-induced inactivation of PDX-1 (Coskun et al., 2005; Baumel-Alterzon and Scott, 2022). In the current study, Ceph significantly decreased hyperglycemia and restored the decrease in plasma insulin levels in diabetic rats. Moreover, Ceph restored PDX-1 expression, maintained integrity of β-cells, and decreased TUNEL^+^ nuclei after STZ treatment. Thus, based on previous findings (Samra et al., 2016), we could postulate that Ceph anti-oxidant effects and its ability to suppress NF-κB activation could function to reduce β-cells apoptosis, preserve PDX-1 expression and maintain β-cells functional integrity, which in turn restores insulin secretion and improves glycemic status in STZ-induced insulin-deficient diabetic rats.

Endothelial dysfunction in diabetes is primarily attributed to increased oxidative stress-induced impairment of NO bioavailability and subsequent reduction of cGMP signaling (Hadi and Suwaidi, 2007; Fernandes-Costa et al., 2024). It is well known that elevation in oxidative stress in cardiovascular disease including diabetes can cause endothelial dysfunction due to increased production of reactive oxygen species, which can lead to depletion of NO as a key vasodilator molecule (Koren et al., 2025). In the current study, STZ-induced diabetic rats showed an increase in plasma TBARs, indicating increased lipid peroxidation and this increase was accompanied by reduction in plasma cGMP levels suggesting a decrease in NO bioavailability which will trigger vascular injury. Vascular injury in diabetic rats translates to endothelial dysfunction since Ach-induced vascular relaxation in aortic rings was impaired in diabetic vs. control rats whereas relaxation responses to SNP remained unaffected, suggesting that STZ-induced insulin-deficient diabetes impairs endothelial function without altering smooth vascular muscle responsiveness. Since Ceph treatment reduced plasma TBARs and improved NO bioavailability in diabetic rats, this could also explain how Ceph improved the impairment in Ach-induced vascular relaxation in aortic rings in diabetic rats. These results indicate that the Ceph preserves NO-cGMP signaling and restores endothelial integrity, thus improving endothelial vascular function in diabetes. These effects aligns with earlier findings which demonstrated that Ceph attenuated oxidative stress, NF-κB activation and vascular inflammation in general (Kudo et al., 2011; PaudelKarki and Kim, 2016), further supporting a crucial role of Ceph in maintaining vascular homeostasis in diabetes.

Previous studies have identified STZ-induced oxidative stress as a key factor in development and progression of diabetic nephropathy (Fernandes et al., 2016; Wang and Zhang, 2024). Oxidative injury and chronic hyperglycemia promote glomerular injury and lead to gradual reduction in podocalyxin expression in podocytes. This is followed by damage to podocyte foot processes and loss of glomerular structural integrity, which impairs glomerular filtration barrier and results in increased urinary protein excretion (Fang et al., 2013; Zhai et al., 2015). Additionally, STZ-induced oxidative stress and injury stimulates ICAM-1 expression to facilitate recruitment of immune cells to the kidney to further aggravate renal inflammation (Elmarakby et al., 2011). Consequently, aberrant inflammation and injury lead to secretion of profibrotic factors such as TGF-β leading to increased extracellular matrix production and pronounced renal fibrosis (Yuan et al., 2022). In the current study, STZ induced significant increases in urinary TBARs and podocalyxin excretion levels together with marked impairment in renal function as evidenced by decreased creatinine clearance and increased albuminuria. Histopathological examination of diabetic kidneys also revealed elevation in collagen deposition and renal fibrosis which is a consequence of severe hyperglycemia and hyperfiltration. The reno-protective effects of Ceph in diabetic rats were evident by the reduction of podocalyxin and albumin excretion levels together with improved creatinine clearance. Furthermore, Ceph reduced renal collagen deposition and collagen IV expression, confirming its antifibrotic activity in diabetic rats. These effects may be also linked to the ability of Ceph to suppress renal oxidative stress and inflammation, as reflected by decreased renal TBARs, ICAM-1, and TGF-β levels in diabetic rats. Our findings agree with previous work where Ceph inhibited NF-κB and NLRP3 inflammasome activation in STZ-induced insulin-deficient diabetic rats (Samra et al., 2016). The decrease in plasma TGF-β in diabetic vs. control rats and the increase in renal TGF-β in diabetic vs. control rats suggest that kidney fibrosis in diabetes is a local process rather than a spill-over from the circulation. The reduction in renal ICAM-1 and TGF-β by Ceph treatment in diabetic rats also suggests that Ceph is effective in halting the progression of renal inflammation and fibrosis in diabetic rats. Overall, given the close interplay between oxidative stress, inflammation, and fibrosis in the incidence and progression of diabetic renal injury, Ceph could act directly to attenuate these pathways or indirectly via improving glycemic status and β-cells function, which likely underlies its ability to halt the progression of diabetic renal injury. However, Ceph-induced improvement in β-cell function might also be secondary to reduction in hyperglycemia-induced oxidative injury and inflammation. Notably, the free radical scavenging property of Ceph stems from its unique bis-benzylisoquinoline structure. Ceph ability to inhibit inflammasome activation and IL-1β release in diabetic rat kidneys (Samra et al., 2016) could be a reasonable mechanism that connect metabolic stress with both β-cell death and renal inflammation in diabetes.

A key limitation of the current study is its reliance on the six-week, sub-chronic STZ-induced insulin deficient diabetes. While this duration is adequate for observing significant organ injury, the findings may not fully translate to the long-term, complex, and multifactorial human disease. We also assessed random blood glucose rather than fasting blood glucose levels which are not typically and commonly used for diabetic rodent models. This is because severe hyperglycemic rats are heavily dependent on food to maintain energy and fasting could exacerbate severe ketoacidosis and animal loss, especially since we did not supplement STZ-diabetic rats with low dose of insulin. Studies have previously reported that Ceph also significantly lowered fasting blood glucose levels in STZ-induced insulin deficient diabetes (Samra et al., 2016). Future directions will focus on assessing oral glucose tolerance curve to undoubtedly confirm that Ceph can improve insulin deficient diabetes. Although findings from previous and current studies support both the direct tissue-protective actions of Ceph. And indirect metabolic benefits, the mechanistic relationships remain primarily correlative. While preservation of pancreatic PDX-1 expression by Ceph treatment in diabetic rats suggests maintenance of β-cell identity, direct functional assessment of insulin secretory capacity was not performed. Future studies will pharmacologically target NF-κB or inflammasome pathways, as well as functionally address metabolic parameters (e.g., glucose tolerance testing) to delineate whether Ceph exerts its protective effects primarily through direct preservation of β-cell function or indirectly via attenuation of oxidative stress and inflammation.

## 5 Conclusions and clinical implications

Collectively, our findings suggest that Ceph exerts reno-protective effects through both direct renal antioxidant and anti-inflammatory effects and/or indirect regulation of glycemic status via improving pancreatic β‐cell function.

While current therapeutic strategies for diabetic complications, such as ACE inhibitors and SGLT2 inhibitors, focus primarily on hemodynamic and metabolic control, Ceph demonstrated a unique cytoprotective effect on pancreatic β-cells and vascular endothelial function and inflammation. Ceph dual metabolic and organ-protective advantages in diabetes make it a possible future valuable adjunctive therapy to insulin for type 1 diabetic patients based on its antioxidant and anti-inflammatory properties that current oral hypoglycemic monotherapies may not target.

## Data Availability

The original contributions presented in the study are included in the article/Supplementary Material, further inquiries can be directed to the corresponding author.

## References

[B1] Ayodele O. E. Alebiosu C. O. Salako B. L. (2004). Diabetic nephropathy--a review of the natural history, burden, risk factors and treatment. J. Natl. Med. Assoc. 96, 1445–1454. 15586648 PMC2568593

[B2] Bailly C. (2019). Cepharanthine: an update of its mode of action, pharmacological properties and medical applications. Phytomedicine 62, 152956. 10.1016/j.phymed.2019.152956 31132753 PMC7126782

[B3] Ban C. R. Twigg S. M. (2008). Fibrosis in diabetes complications: pathogenic mechanisms and circulating and urinary markers. Vasc. Health Risk Manag. 4, 575–596. 10.2147/vhrm.s1991 18827908 PMC2515418

[B4] Baumel-Alterzon S. Scott D. K. (2022). Regulation of Pdx1 by oxidative stress and Nrf2 in pancreatic beta-cells. Front. Endocrinol. (Lausanne) 13, 1011187. 10.3389/fendo.2022.1011187 36187092 PMC9521308

[B5] Coskun O. Kanter M. Korkmaz A. Oter S. (2005). Quercetin, a flavonoid antioxidant, prevents and protects streptozotocin-induced oxidative stress and beta-cell damage in rat pancreas. Pharmacol. Res. 51, 117–123. 10.1016/j.phrs.2004.06.002 15629256

[B6] Donate-Correa J. Ferri C. M. Sanchez-Quintana F. Perez-Castro A. Gonzalez-Luis A. Martin-Nunez E. (2020). Inflammatory cytokines in diabetic kidney disease: Pathophysiologic and therapeutic implications. Front. Med. (Lausanne) 7, 628289. 10.3389/fmed.2020.628289 33553221 PMC7862763

[B7] Einarson T. R. Acs A. Ludwig C. Panton U. H. (2018). Prevalence of cardiovascular disease in type 2 diabetes: a systematic literature review of scientific evidence from across the world in 2007-2017. Cardiovasc Diabetol. 17, 83. 10.1186/s12933-018-0728-6 29884191 PMC5994068

[B8] Elmarakby A. A. Sullivan J. C. (2012). Relationship between oxidative stress and inflammatory cytokines in diabetic nephropathy. Cardiovasc. Ther. 30, 49–59. 10.1111/j.1755-5922.2010.00218.x 20718759

[B9] Elmarakby A. A. Abdelsayed R. Yao Liu J. Mozaffari M. S. (2010). Inflammatory cytokines as predictive markers for early detection and progression of diabetic nephropathy. Epma J. 1, 117–129. 10.1007/s13167-010-0004-7 23199046 PMC3405301

[B10] Elmarakby A. A. Ibrahim A. S. Faulkner J. Mozaffari M. S. Liou G. I. Abdelsayed R. (2011). Tyrosine kinase inhibitor, genistein, reduces renal inflammation and injury in streptozotocin-induced diabetic mice. Vasc. Pharmacol. 55, 149–156. 10.1016/j.vph.2011.07.007 21807121

[B11] Elmarakby A. A. Faulkner J. Baban B. Saleh M. A. Sullivan J. C. (2012). Induction of hemeoxygenase-1 reduces glomerular injury and apoptosis in diabetic spontaneously hypertensive rats. Am. J. Physiology-Renal Phys. 302, F791–F800. 10.1152/ajprenal.00472.2011 22205229 PMC3340931

[B12] Fang J. Wei H. Sun Y. Zhang X. Liu W. Chang Q. (2013). Regulation of podocalyxin expression in the kidney of streptozotocin-induced diabetic rats with Chinese herbs (yishen capsule). BMC Complement. Altern. Med. 13, 76. 10.1186/1472-6882-13-76 23560927 PMC3637235

[B13] Fernandes S. M. Cordeiro P. M. Watanabe M. Fonseca C. D. Vattimo M. F. (2016). The role of oxidative stress in streptozotocin-induced diabetic nephropathy in rats. Arch. Endocrinol. Metab. 60, 443–449. 10.1590/2359-3997000000188 27812607 PMC10118643

[B14] Fernandes-Costa F. Gomes Da Silva R. T. De Almeida A. J. P. O. De Medeiros I. A. De Assis Tafuri L. S. Dos Santos G. J. (2024). Organic vs. inorganic nitrates: Metabolic and vascular outcomes in STZ-Induced diabetes in mice. Life Sci. 359, 123257. 10.1016/j.lfs.2024.123257 39561873

[B15] Garofalo C. Borrelli S. Liberti M. E. Andreucci M. Conte G. Minutolo R. (2019). SGLT2 inhibitors: nephroprotective efficacy and side effects. Medicina 55, 268. 10.3390/medicina55060268 31212638 PMC6630922

[B16] Ghasemi A. Jeddi S. (2023). Streptozotocin as a tool for induction of rat models of diabetes: a practical guide. Excli J. 22, 274–294. 10.17179/excli2022-5720 36998708 PMC10043433

[B17] Hadi H. A. Suwaidi J. A. (2007). Endothelial dysfunction in diabetes mellitus. Vasc. Health Risk Manag. 3, 853–876. 18200806 PMC2350146

[B18] Jin Q. Liu T. Qiao Y. Liu D. Yang L. Mao H. (2023). Oxidative stress and inflammation in diabetic nephropathy: role of polyphenols. Front. Immunol. 14, 1185317. 10.3389/fimmu.2023.1185317 37545494 PMC10401049

[B19] Koren L. Koren A. Lojo N. Likic R. Haluzan D. Crkvenac Gregorek A. (2025). Spotlight commentary: nitric oxide, vascular dysfunction and emerging therapeutic insights. Br. J. Clin. Pharmacol. 92 (2), 348–350. 10.1002/bcp.70383 41292098

[B20] Kudo K. Hagiwara S. Hasegawa A. Kusaka J. Koga H. Noguchi T. (2011). Cepharanthine exerts anti-inflammatory effects Via NF-κB inhibition in a LPS-induced rat model of systemic inflammation. J. Surg. Res. 171, 199–204. 10.1016/j.jss.2010.01.007 20334881

[B21] Li X. Wu Y. Song Y. Ding N. Lu M. Jia L. (2020). Activation of NF-κB-Inducing kinase in islet β cells causes β cell failure and diabetes. Mol. Ther. 28, 2430–2441. 10.1016/j.ymthe.2020.07.016 32730745 PMC7647925

[B22] Liu D. Liu Z. (2020). Atrasentan in patients with diabetes and chronic kidney disease. Lancet 395, 269–270. 10.1016/S0140-6736(19)33021-1 31982064

[B23] Liu K. Hong B. Wang S. Lou F. You Y. Hu R. (2023). Pharmacological activity of cepharanthine. Molecules 28, 5019. 10.3390/molecules28135019 37446681 PMC10343550

[B24] Paudel K. R. Karki R. Kim D.-W. (2016). Cepharanthine inhibits *in vitro* VSMC proliferation and migration and vascular inflammatory responses mediated by RAW264.7. Toxicol. Vitro 34, 16–25. 10.1016/j.tiv.2016.03.010 27021874

[B25] Rogosnitzky M. Danks R. (2011). Therapeutic potential of the biscoclaurine alkaloid, cepharanthine, for a range of clinical conditions. Pharmacol. Rep. 63, 337–347. 10.1016/s1734-1140(11)70500-x 21602589

[B26] Rout P. Jialal I. (2025). Diabetic nephropathy, in Statpearls. (Treasure Island (FL): StatPearls Publishing Copyright © 2025, StatPearls Publishing LLC.).

[B27] Ruiz-Ortega M. Lamas S. Ortiz A. (2022). Antifibrotic agents for the management of CKD: a review. Am. J. Kidney Dis. 80, 251–263. 10.1053/j.ajkd.2021.11.010 34999158

[B28] Saad K. M. Salles E. L. Naeini S. E. Baban B. Abdelmageed M. E. Abdelaziz R. R. (2024). Reno-protective effect of protocatechuic acid is independent of sex-related differences in murine model of UUO-Induced kidney injury. Pharmacol. Rep. 76, 98–111. 10.1007/s43440-023-00565-2 38214881

[B29] Samra Y. A. Said H. S. Elsherbiny N. M. Liou G. I. El-Shishtawy M. M. Eissa L. A. (2016). Cepharanthine and piperine ameliorate diabetic nephropathy in rats: role of NF-κB and NLRP3 inflammasome. Life Sci. 157, 187–199. 10.1016/j.lfs.2016.06.002 27266851

[B30] Shahbazian H. Rezaii I. (2013). Diabetic kidney disease; review of the current knowledge. J. Ren. Inj. Prev. 2, 73–80. 10.12861/jrip.2013.24 25340133 PMC4206005

[B31] Tonneijck L. Muskiet M. H. Smits M. M. Van Bommel E. J. Heerspink H. J. Van Raalte D. H. (2017). Glomerular hyperfiltration in diabetes: mechanisms, clinical significance, and treatment. J. Am. Soc. Nephrol. 28, 1023–1039. 10.1681/ASN.2016060666 28143897 PMC5373460

[B32] Wang N. Zhang C. (2024). Oxidative stress: a culprit in the progression of diabetic kidney disease. Antioxidants 13, 455. 10.3390/antiox13040455 38671903 PMC11047699

[B33] Wang Q. Mergia E. Koesling D. Mittmann T. (2017). Nitric oxide/cGMP signaling via guanylyl cyclase isoform 1 modulates glutamate and GABA release in somatosensory cortex of mice. Neuroscience 360, 180–189. 10.1016/j.neuroscience.2017.07.063 28782641

[B34] Yang D. R. Wang M. Y. Zhang C. L. Wang Y. (2024). Endothelial dysfunction in vascular complications of diabetes: a comprehensive review of mechanisms and implications. Front. Endocrinol. (Lausanne) 15, 1359255. 10.3389/fendo.2024.1359255 38645427 PMC11026568

[B35] Yau K. Dharia A. Alrowiyti I. Cherney D. Z. I. (2022). Prescribing SGLT2 inhibitors in patients with CKD: expanding indications and practical considerations. Kidney Int. Rep. 7, 1463–1476. 10.1016/j.ekir.2022.08.016 35812300 PMC9263228

[B36] Yuan Y. Liu Y. Sun M. Ye H. Feng Y. Liu Z. (2022). Aggravated renal fibrosis is positively associated with the activation of HMGB1-TLR2/4 signaling in STZ-Induced diabetic mice. Open Life Sci. 17, 1451–1461. 10.1515/biol-2022-0506 36448056 PMC9658007

[B37] Zeng L. Szeto C. C. (2021). Urinary podocyte markers in kidney diseases. Clin. Chim. Acta 523, 315–324. 10.1016/j.cca.2021.10.017 34666027

[B38] Zhai L. Gu J. Yang D. Wang W. Ye S. (2015). Metformin ameliorates podocyte damage by restoring renal tissue podocalyxin expression in type 2 diabetic rats. J. Diabetes Res. 2015, 231825. 10.1155/2015/231825 26075281 PMC4444588

