# Supplementary figures and images for: Cepharanthine improves blood glucose levels and mitigates renal injury in streptozotocin-induced diabetic rats via restoring pancreatic β-cell integrity and reducing inflammation
Source: Front Pharmacol. 2026 Jun 19;17:1807706. doi: 10.3389/fphar.2026.1807706 (PMC13328083; doi:10.3389/fphar.2026.1807706)

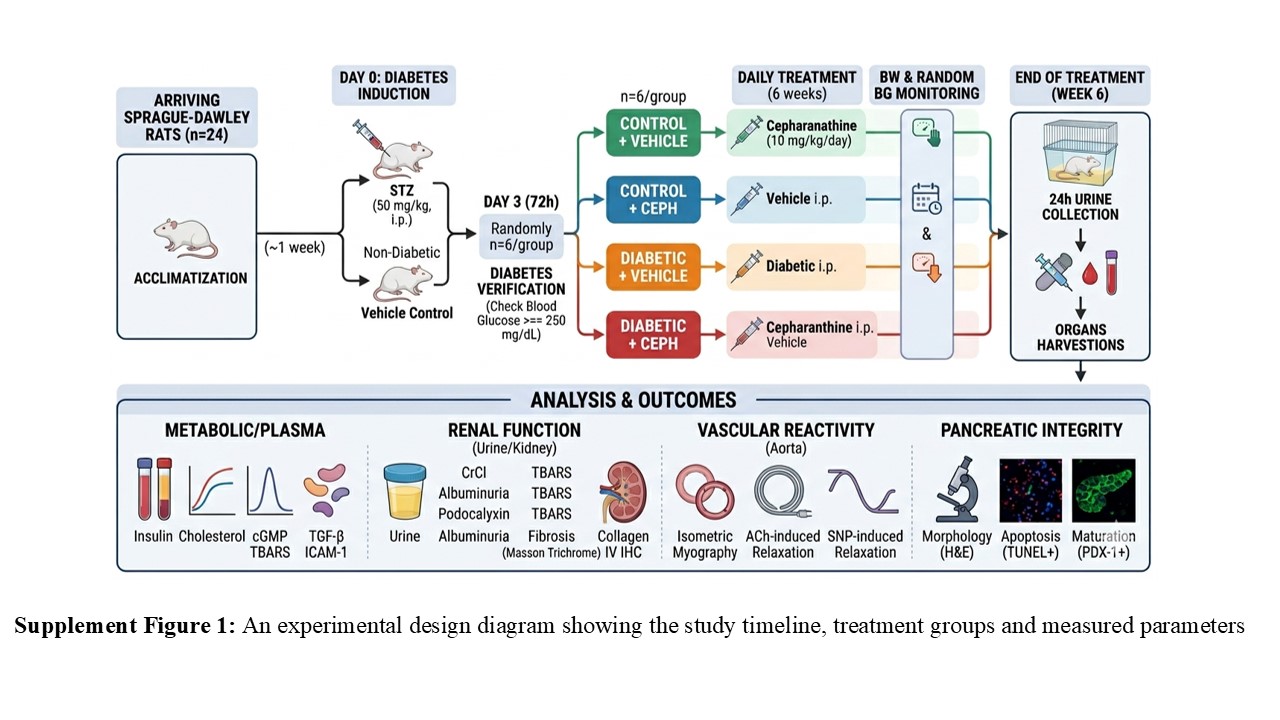

Supplement: Supplementary file 1 [file Image1.jpeg]

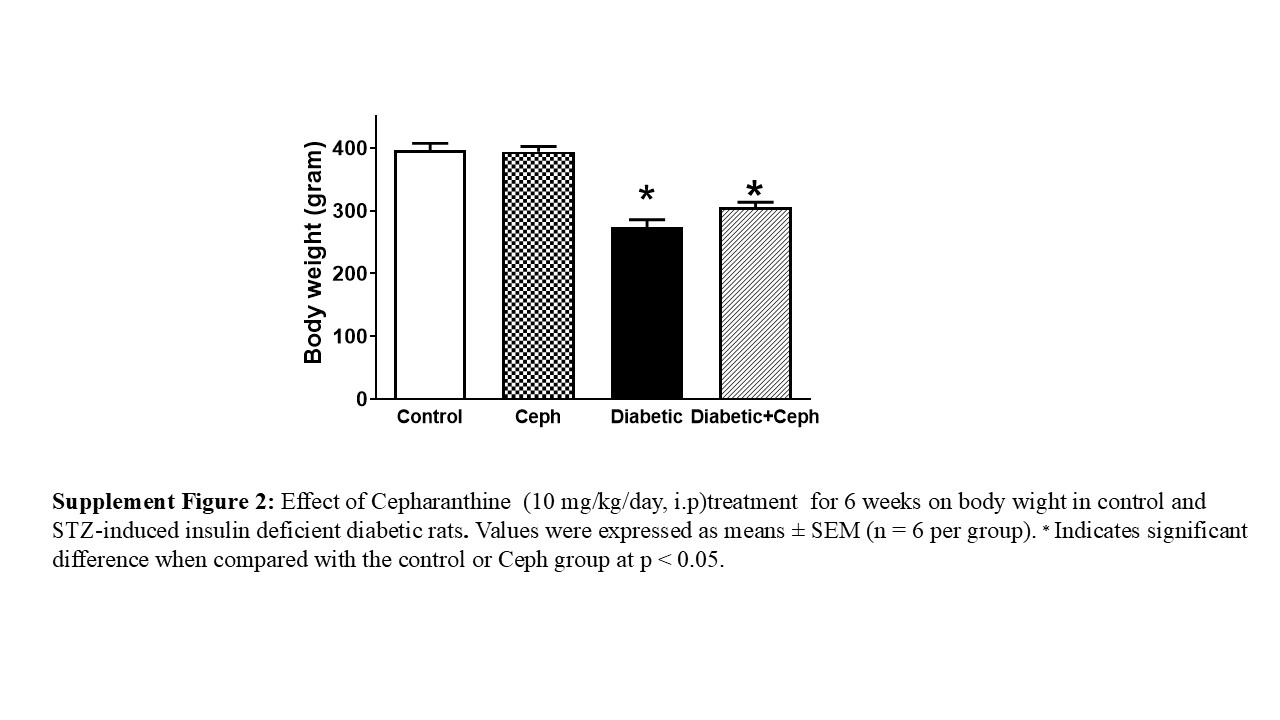

Supplement: Supplementary file 2 [file Image2.jpeg]
